# Patient characteristics, vital signs and tcpCO_2_ dynamics significantly influence tcpCO_2_ accuracy in extremely preterm infants: an observational study

**DOI:** 10.3389/fped.2026.1878177

**Published:** 2026-07-02

**Authors:** Lena Olivier, Camelia Lauterbach Oprea, André Stollenwerk, Valerie Pfannschmidt, Thorsten Orlikowsky, Mark Schoberer

**Affiliations:** 1 Division of Neonatology, Department of Pediatric and Adolescent Medicine, Uniklinik RWTH Aachen, Aachen, Germany; 2 Chair of Embedded Software (Computer Science 11), RWTH Aachen University, Aachen, Germany

**Keywords:** extremely premature infant, neonatal intensive care unit, neonates, tcPCO_2_, transcutaneous CO2

## Abstract

**Purpose:**

The estimation of the partial pressure of carbon dioxide (pCO_2_) is of interest in extremely premature infants. Neonatal intensive care units use transcutaneous pCO_2_ monitoring (tcpCO_2_) as a trend parameter and continuous estimate of pCO_2_. Our objective was to identify candidate influence factors on the accuracy of tcpCO_2_ in extremely premature infants in their first week of life.

**Methods:**

In a retrospective cohort study, linear mixed-effects models were used to analyze the influence of patient characteristics, vital signs and tcpCO_2_ dynamics on the difference of tcpCO_2_ and capillary pCO_2_ as reference (ΔpCO_2_). Candidate parameters identified in univariate analyses were incorporated into multivariable models to confirm their effect on ΔpCO_2_.

**Results:**

500 measurements from 29 extremely preterm infants were analyzed. Postnatal age, mean fraction of inspired oxygen, tcpCO_2_, capillary pCO_2_, last tcpCO_2_ and last ΔpCO_2_ were positively associated with ΔpCO_2_, while gestational age and hemoglobin were negatively associated with ΔpCO_2_.

**Conclusion:**

These candidate parameters may be used to enhance the non-invasive estimation of pCO_2_ from tcpCO_2_ in the future.

**Trial registration:**

German Clinical Trials Register (DRKS00033228, first registered 29 August 2024, https://drks.de/search/en/trial/DRKS00033228/details)

## Introduction

Prematurity is defined as birth before 37 weeks of gestation and affects approximately 10% of neonates (1). Extremely premature infants (EPIs, < 28 weeks of gestation) constitute 4.2% of all premature infants (2). Despite medical progress for this patient group, mortality is still high (3). The first week of life is particularly critical as a large proportion of complications and deaths occur at this stage (4). EPIs are confronted with various complications concerning all organ systems (5). Especially respiratory failure is a common challenge, which often requires intubation and mechanical ventilation to maintain gas exchange (6).

Episodes of hypo- and hypercapnia as well as large fluctuations of the partial pressure of carbon dioxide (pCO_2_) are known to influence the cerebral perfusion (7). They can hereby contribute to cerebral pathologies such as periventricular leukomalacia (8) and germinal matrix bleedings (9, 10). Severe neurodevelopmental impairment can result from these conditions and entail life-long implications (11–13). The tight monitoring of pCO_2_ is therefore of particular interest.

Blood gas analyses (BGA) provide the gold standard of pCO_2_ measurement (14). However, they are invasive, cause patient stress, blood loss and often impede minimal handling strategies (15, 16). Transcutaneous pCO_2_ measurements (tcpCO_2_) can provide a continuous estimate of pCO_2_ and are therefore frequently used on neonatal intensive care units (NICUs) (17). tcpCO_2_ measurement is conducted by a sensor which is applied to the skin. It heats up to induce a local hyperemia to increase capillary perfusion and then quantifies the amount of CO_2_, which passes the skin via diffusion (18).

A reliable estimate of pCO_2_ from non-invasive pCO_2_ measurement techniques has the potential to reduce the number of conducted BGAs and decrease patient stress (19). Previous work showed that tcpCO_2_ monitoring is feasible in premature infants (20) and is associated with fewer episodes of hypo- and hypercarbia (21). A moderate correlation of tcpCO_2_ and pCO_2_ and their trends was found in premature infants < 1,000 g, regardless of their day of life (22, 23). Apart from device-specific factors such as calibration, sensor location and temperature (24), patient-specific factors can influence the deviation of tcpCO_2_ from pCO_2_ (ΔpCO_2_). In a group of EPIs, a large inter-individual variation was observed concerning the agreement of pCO_2_ and tcpCO_2_ (22). Van Essen et al. showed that postnatal age, arterial systolic blood pressure, body temperature and arterial partial pressure of oxygen influence ΔpCO_2_ in premature neonates (median gestational age: 27 3/7 weeks) (25). These results demonstrate that tcpCO_2_ alone cannot be regarded as a non-invasive surrogate parameter of pCO_2_. A variety of additional clinical information is useful to enhance the interpretation of tcpCO_2_ and estimation of pCO_2_.

## Objective

EPIs constitute one of the most vulnerable patient groups in neonatology. To the best of our knowledge, possible factors influencing the accuracy of tcpCO_2_ have not yet extensively been evaluated in this patient group. Our objective was to analyze the impact of vital signs, tcpCO_2_ dynamics and patient characteristics on ΔpCO_2_ in EPIs in their first week of life.

## Materials and methods

### Study design

The study was conducted as a retrospective observational cohort study. Data collection was performed between 29 August 2024 and 30 April 2026.

### Ethical approval

The overall project AIx-Neo-Guard was approved by the local ethics committee [EK 24-213, Ethik-Kommission an der Medizinischen Fakultät der Rheinisch-Westfälischen Technischen Hochschule Aachen (RWTH Aachen)] and prospectively registered at the German Clinical Trials Register (DRKS00033228). Exemption from informed parental consent was granted by the German Health Data Utilization Act because only data used for diagnostics and treatment were collected. Parents could revoke study participation at any time. The analysis was conducted in accordance with the Declaration of Helsinki (26) and the “The Strengthening the Reporting of Observational Studies in Epidemiology (STROBE) statement: guidelines for reporting observational studies” guideline (27).

### Setting

Data were collected on the tertiary care NICU of Uniklinik RWTH Aachen, Germany. The ward provides 9 beds, each equipped to provide neonatal respiratory support. Approximately 20 EPIs are treated on the NICU annually.

### Participants

Patients were included in the analysis if they met all the following inclusion criteria:

- born extremely premature (< 28 + 0 weeks of gestation)
- treated on the NICU in their first week of life
- both tcpCO_2_ monitoring and intermittent capillary pCO_2_ (pcCO_2_) measurements were conducted
- available vital sign monitoring

Patients were excluded if they suffered from hemodynamic shock (defined as persistent mean arterial pressure lower than gestational age with the need for vasopressor therapy), had anasarca or other impairment of the skin, or if parents objected to study participation. Moreover, technical failure of data collection could also lead to exclusion.

### Variables

pcCO_2_ and tcpCO_2_ were used to calculate ΔpCO_2_ according to (1).

(1) ΔpCO_2_ = tcpCO_2_—pcCO_2_

We evaluated the influence of patient characteristics, vital signs, fraction of inspired oxygen (FiO_2_), hemoglobin (Hb), pcCO_2_ and tcpCO_2_ dynamics on ΔpCO_2_ in the patients' first week of life.

Patient characteristics were:

- gestational age (GA)
- birth weight (BW)
- BW percentile
- postnatal age (PNA)
- sex
- early-onset bacterial infections (EOBI).

Regarded vital signs were

- heart rate (HR)
- oxygen saturation (SpO_2_)
- body temperature (Temp).

As tcpCO_2_ dynamics we analyzed:

- current and last tcpCO_2_ value
- last ΔpCO_2_
- time since last tcpCO_2_ measurement.

### Data sources

pcCO_2_ and Hb were quantified using an ABL90 FLEX Plus analyzer (Radiometer GmbH, Krefeld, Germany). GA, PNA, BW and BW percentile were extracted manually from the local patient data management system. tcpCO_2_ monitoring was conducted using a Sentec Digital Monitor with V-Sign^TM^ sensor (SENTEC AG, Therwil, Switzerland). The sensor was applied to the patients' thigh, back, or chest and heated up to a temperature of 40.2 °C according to local standard care. Recalibration was performed during nursing, when required by the device's algorithm or if the correlation of tcpCO_2_ and pcCO_2_ was assessed to be low by the medical staff (e.g., if ΔpCO_2_ was increasing and exceeded 20 mmHg). tcpCO_2_ measurements during the sensor's stabilization phase after calibration were excluded from the analysis. Temp was measured continuously by a skin or rectal probe. HR, SpO_2_ and Temp were monitored by Philips IntelliVue X2 monitors (Philips GmbH Market DACH, Hamburg, Germany). Their values were averaged (mean value) over 5 min to reduce the influence of outliers. All patients received respiratory support by a Leoni plus ventilator (Löwenstein Medical, Bad Ems, Germany), from which FiO_2_ was extracted and averaged over 5 min. All parameter values closest to the respective pcCO_2_ value were used for analysis.

### Bias

We used the same type of measurement devices for each patient according to local standard. Study participation did not influence diagnostics or treatment.

### Study size

All eligible patients were included in the analysis.

### Statistical analysis

Missing data were not imputed. Times of pcCO_2_ measurements without a corresponding tcpCO_2_ value within a window of tolerance of ± 2 min were excluded from the analysis. This window of tolerance was chosen because it is in accordance with typical tcpCO_2_ reaction times to pCO_2_ changes (28, 29) and to account for stabilization times of the used tcpCO_2_ sensors.

Linear mixed-effects models were used to assess associations between each potential influencing factor and ΔpCO_2_. Linear mixed-effects models were chosen to account for varying numbers of measurements per patient (1–34 measurements). The anonymized patient ID was included as a random intercept to account for inter-individual variability in baseline ΔpCO_2_ levels. Fixed effects were entered separately for each potential influencing factor (univariable models). The models were estimated using restricted maximum likelihood. Model estimates (β) with 95% confidence intervals were calculated.

To identify independent predictors of ΔpCO_2_ while accounting for potential confounding, multivariable linear mixed-effects models were constructed. Although a large number of measurements were available, these observations were clustered within a small number of patients. Given the limited number of independent subjects and the potential for overfitting, we did not include all candidate predictors in a single multivariable mixed-effects model. Instead, candidate variables identified in the univariable analyses were grouped into clinically related domains and entered into five separate multivariable models based on *a priori* physiological considerations.

Statistical significance was set at a two-tailed 0.05 level. Analyses were performed using IBM SPSS Statistics version 29.0.0.0 (IBM, Armonk, NY, USA).

## Results

### Participants

35 EPIs were treated on the ward during the study period. Of these, 6 had to be excluded from analysis (anasarca: 1, hemodynamic shock: 1, PNA > 7 days: 3, technical failure: 1), resulting in 29 patients with a total of 500 measurements for analysis. Patient characteristics are shown in Table 1.

**Table 1:** Patient characteristics [shown as mean ± standard deviation (minimum—maximum) where appropriate].

Patient characteristic	mean
Number of individuals	29
Number of measurements	500
GA (weeks)	25.4 ± 1.4 [23–27.86]
BW (grams)	784 ± 194 [410–1,115]
BW percentile	35 ± 21 [2–71]
Male sex (%)	51.7
EOBI within the first week [*n*, (%)]	9 (31.0%)
Required intubation in first week [*n*, (%)]	14 (48.3%)
Death before discharge [*n*, (%)]	3 (10.3%)

### Main analysis

**Figure 1:**
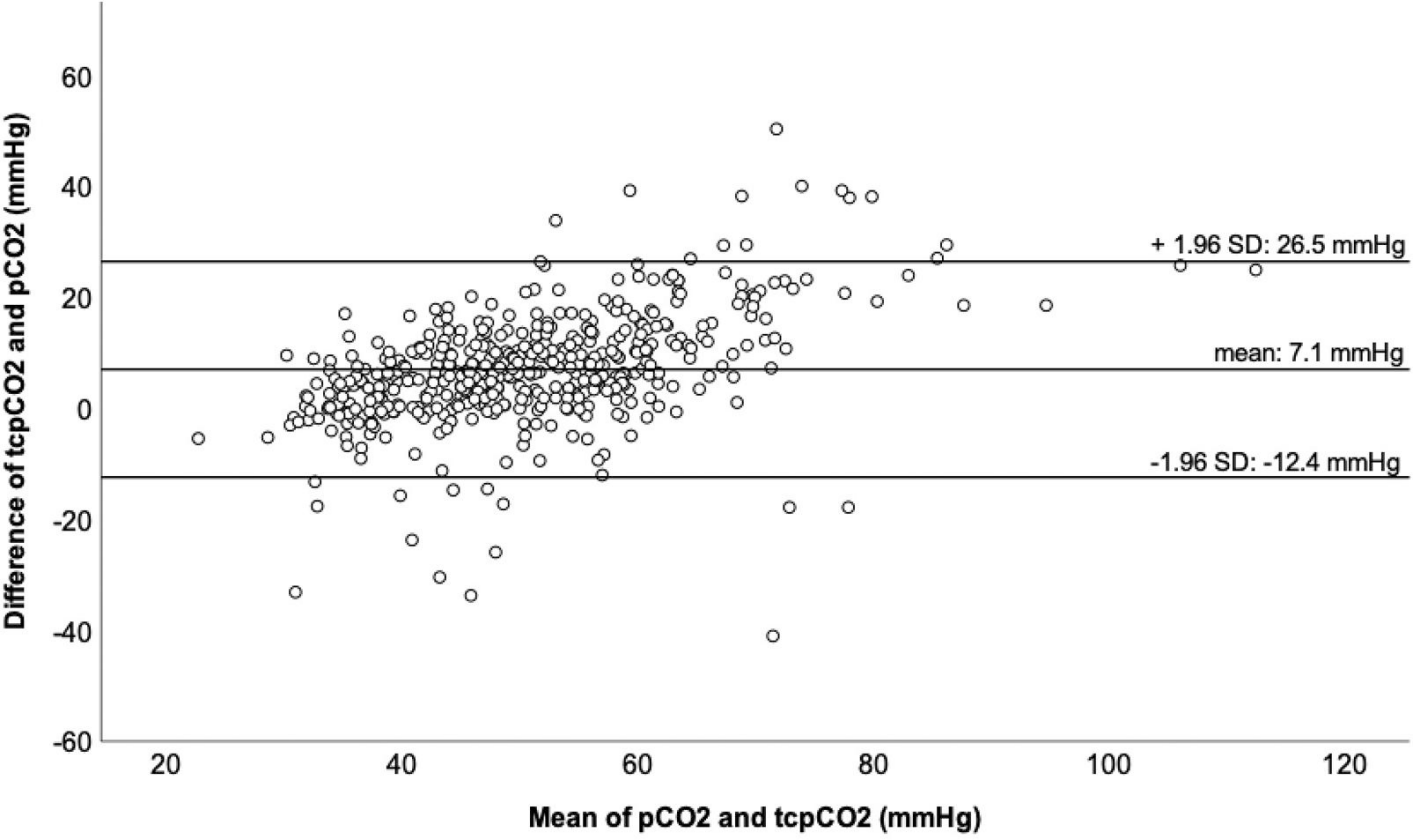
Bland-Altman plot of tcpCO_2_ and pcCO_2_ measurements.

A Bland-Altman plot is shown in Figure 1. The mean difference between tcpCO_2_ and pcCO_2_ was 7.1 mmHg (95% confidence interval 6.2–7.9 mmHg). The limits of agreement ranged from −12.4 to 26.5 mmHg.

Regression analysis revealed a significant proportional bias (*β*1 = 0.42, *p* < 0.001), indicating that ΔpCO_2_ increased with higher measurement levels. To further investigate the proportional bias identified in the Bland–Altman analysis, an additional linear mixed-effects model was performed with mean pCO_2_ [(tcpCO_2_ + pcCO_2_)/2] as a fixed effect. Mean pCO_2_ was significantly associated with ΔpCO_2_ (*β*1 = 0.42, *p* < 0.001), indicating that the difference between tcpCO_2_ and pCO_2_ increased with increasing CO_2_ levels.

In the univariable linear mixed-effects model, GA, PNA, BW, sex, Hb, mean SpO_2_, mean FiO_2_, pcCO_2_, tcpCO_2_, last tcpCO_2_ and last ΔpCO_2_ were significantly associated with ΔpCO_2_ (Table 2). The fixed-effects estimates *β*1 represent the population-level effect of the tested parameters on ΔpCO_2_.

**Table 2:** Results from linear mixed-effects models to test association with ΔpCO_2_.

Parameter	Estimate (*ß*1)	95% confidence interval for *ß*1	*p*-value
GA (weeks)	−1.57	−2.15; −0.99	**<0** **.** 001*
PNA (days)	1.36	0.91; 1.82	**<0**.001*
BW (grams)	−0.005	−0.01; −0.001	**0**.**012**
BW percentile	−0.011	−0.05; 0.03	0.565
Sex (female)	−4.77	−6.50; −3.05	**<0**.001*
EOBI (non-EOBI)	−1.07	−2.88; 0.74	0.246
Mean HR (/min)	0.04	−0.02; 0.11	0.2
Mean SpO_2_ (%)	−0.29	−0.44; −0.14	**<0**.001*
Mean Temp ( °C)	−1.42	−3.47; 0.64	0.176
Mean FiO_2_ (%)	0.34	0.2; 0.48	<0.001*
Hb (g/dL)	−0.75	−1.14; −0.35	<0.001*
pcCO_2_ (mmHg)	0.09	0.06; 0.18	**0**.**036**
tcpCO_2_ (mmHg)	0.48	0.45; 0.53	<0.001*
Last tcpCO_2_ (mmHg)	0.29	0.24; 0.35	<0.001*
Last ΔpCO_2_ (mmHg)	0.49	0.42; 0.57	<0.001*
Time since last BGA (hours)	−3,48e−5	−4.14e−5; 3.44e−5	0.857

Statistical significances are highlighted by bold values.

**Figure 2:**
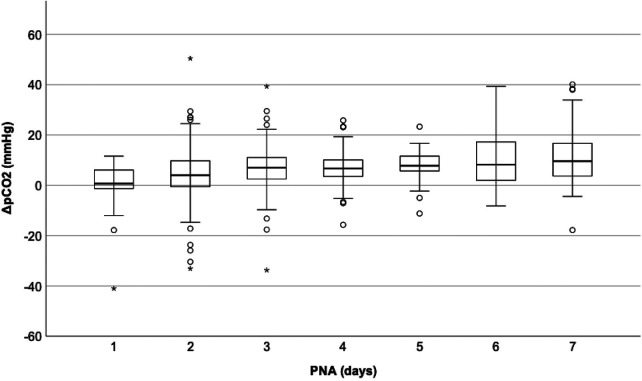
ΔpCO_2_ depending on postnatal age.

Of these, PNA (Figure 2), mean FiO_2_, pcCO_2_, tcpCO_2_, last tcpCO_2_ and last ΔpCO_2_ were positively associated with ΔpCO_2_ and GA, BW, female sex, mean SpO_2_ and Hb were negatively associated.

Male sex had significantly higher ΔpCO_2_ compared to female sex (9.1 ± 9.7 mmHg vs. 4.3 ± 9.6 mmHg, *p* < 0.001). No significant association was observed for BW percentile, EOBI, mean HR, mean Temp, pcCO_2_ and time since last BGA.

To identify independent predictors of ΔpCO_2_ and account for potential confounding, five multivariable linear mixed-effects models were constructed based on predefined clinical and physiological considerations. The models assessed the independent associations of (1) GA and BW, (2) PNA and Hb, (3) GA and Hb (4) oxygenation-related variables (mean SpO_2_ and FiO_2_), and (5) sex, mean FiO_2_, pcCO_2_, and tcpCO_2_. Regression coefficients (*β*1), 95% confidence intervals (CI), and *p*-values are shown in Table 3.

1. GA was independently associated with ΔpCO_2_ after adjustment for BW. Each additional week of gestation was associated with a decrease of 2.16 mmHg in ΔpCO_2_ (*β*1 = −2.16, *p* < 0.001). BW was not independently associated with ΔpCO_2_ after adjustment for GA (*β*1 = 0.01, *p* = 0.059).
2. Both PNA and Hb were independently associated with ΔpCO_2_. Increasing PNA was associated with higher ΔpCO_2_ values (*β*1 = 1.47, *p* < 0.001), whereas higher Hb was associated with lower ΔpCO_2_ values (*β*1 = −0.72, *p* < 0.001).
3. Both GA and Hb were independently associated with ΔpCO_2_. Increasing GA and Hb were both negatively associated with ΔpCO_2_ (*β*1 = −1.47, *p* < 0.001 and *β*1 = −0.66, *p* < 0.001).
4. Mean FiO_2_ was independently associated with ΔpCO_2_ (*β*1 = 0.33, *p* < 0.001), whereas mean SpO_2_ was not significantly associated with ΔpCO_2_ after adjustment for FiO_2_ (*β*1 = −0.10, *p* = 0.286).
5. Mean FiO_2_, pcCO_2_, and tcpCO_2_ were independently associated with ΔpCO_2_. Higher mean FiO_2_ was associated with a small decrease in ΔpCO_2_ (*β*1 = −0.03, *p* = 0.019). pcCO_2_ was negatively associated with ΔpCO_2_ (*β*1 = −0.99, *p* < 0.001), whereas tcpCO_2_ was positively associated with ΔpCO_2_ (*β*1 = 1.01, *p* < 0.001). Sex was not independently associated with ΔpCO_2_ (*p* = 0.887).

**Table 3:** Results from the multivariable linear mixed-effects models.

Multivariable model	Parameter	Estimate (*ß*1)	95% confidence interval for *ß*1	*p*-value
GA and BW	GA (weeks)	−2.16	−3.00; 1.32	**<0.001**
	BW (grams)	0.01	0.00; 0.01	0.059
Hb and PNA	Hb (g/dL)	−0.72	−1.10; −0.34	**<0.001**
	PNA (days)	1.47	0.98; 1.96	**<0.001**
Hb and GA	Hb (g/dL)	−0.66	−1.05; −0.28	**<0.001**
	GA (weeks)	−1.47	−2.07; −0.87	**<0.001**
FiO_2_ and SpO_2_	FiO_2_: (%)	0.33	0.18; 0.47	**<0.001**
	SpO_2_: (%)	−0.10	−0.28; 0.08	0.286
Sex, FiO*_2_*, tcpCO*_2_*, pcCO*_2_*	sex	0.03	−0.34; 0.39	0.887
	FiO_2_: (%)	−0.03	−0.06; −0.01	**0.019**
	tcpCO_2_: (mmHg)	1.01	0.99; 1.03	**<0.001**
	pcCO_2_: (mmHg)	−0.99	−1.01; −0.97	**<0.001**

Statistical significances are highlighted by bold values.

## Discussion

### Key results

In this study, we evaluated potential influence factors on ΔpCO_2_ in EPIs in their first week of life. In univariate analyses, GA, PNA, BW, sex, Hb, mean SpO_2_, mean FiO_2_, pcCO_2_, tcpCO_2_, last tcpCO_2_ and last ΔpCO_2_ were significantly associated with ΔpCO_2_. Multivariable mixed-effects analyses demonstrated that not all associations observed in the univariable analyses were independent. After adjustment for clinically related covariates, BW, mean SpO_2_, and sex were no longer significantly associated with ΔpCO_2_, whereas GA, PNA, Hb, FiO_2_, pcCO_2_, and tcpCO_2_ remained significant independent candidate predictors.

These findings support the hypothesis that multiple patient- and measurement-related factors contribute to the difference between tcpCO_2_ and pcCO_2_ measurements and should be considered when using tcpCO_2_ as a non-invasive estimate of pCO_2_.

### Interpretation

CO_2_ monitoring and management is highly relevant in EPIs due to the association of pCO_2_ extremes and fluctuations with cerebral pathologies (30). An improvement of non-invasive pCO_2_ estimation is desirable because it can decrease the number of needed BGAs and hereby reduce loss of blood and patient stress of the most vulnerable patients. This, in turn, is well compatible with minimal handling strategies. Former studies showed that the use of tcpCO_2_ monitoring can reduce the incidence of hypo- and hypercapnic episodes, which also led to a lower incidence of germinal matrix bleedings (21). We believe that a more reliable estimate of pCO_2_ can further enhance this effect. The reported accuracy of tcpCO_2_ is, however, controversial (31–33). Therefore, tcpCO_2_ monitoring currently constitutes a useful complementary trend parameter (34).

In this work, we could demonstrate that tcpCO_2_ should be regarded in consideration of the influencing factors to ΔpCO_2_. This information can be used to improve the non-invasive estimation of pCO_2_. Sandberg et al. previously found a small effect of BW and PNA on ΔpCO_2_ (31). ΔpCO_2_ was higher in patients with a BW < 1 kg and with a PNA > 7 days, while FiO_2_ did not influence ΔpCO_2_. We could reproduce the effect of a higher PNA and lower BW on ΔpCO_2_, although the effect of BW was no longer significant after adjustment for GA.

We found a negative association between GA and ΔpCO_2_ which may be explained by the immaturity of the skin. The maturity of the skin is directly proportional to the gestational age (35) and is considered completed at 34 weeks of gestation (36). The immature epidermis of EPIs facilitates the diffusion of CO_2_ through the skin. This could explain why higher tcpCO_2_ values are observed with lower gestational age, which in turn increases ΔpCO_2_. However, this observation appears to conflict with the effect of higher PNA on ΔpCO_2_. We would expect postnatal skin maturation to decrease tcpCO_2_ values through the thickening of the skin barrier. The barrier function of the epidermis improves substantially within the first postnatal weeks but is known to remain impaired compared to term infants (36). Despite these maturation processes, the skin of EPIs remains sufficiently permeable throughout the first postnatal week, which might explain why other factors driving ΔpCO_2_ dominate the PNA effect. The design of this study does not allow for a detailed exploration and explanation of this phenomenon, though it would be a matter of interest.

The univariate linear mixed-effects model showed a higher ΔpCO_2_ observed in male EPIs. This effect could not be reproduced in the multivariable model using FiO_2_, tcpCO_2_ and pcCO_2_ as covariates. These parameters were chosen because male infants are known to be more susceptible to complications of prematurity (“male disadvantage hypothesis”) (37). The multivariable analysis supports that male infants may have been more severely affected by respiratory impairment (i.e., required higher FiO_2_, exhibited higher tcpCO_2_ and pcCO_2_), which led to the differences observed in the univariable model.

We could not find a significantly higher ΔpCO_2_ in the EOBI group. All included patients with EOBI presented with clinical symptoms (e.g., respiratory failure) and elevated infection parameters (C—reactive protein). However, the blood culture remained sterile in all patients. In manifest blood culture positive sepsis and larger study cohorts, the differences might have been more pronounced. The differences may be caused by impaired capillary perfusion and arterial hypotonia due to the infection (38). These lead to a delay in CO_2_ transport and elimination from the tissue and hereby increase pCO_2_ (39). The analysis of capillary refill time and blood pressures would have been interesting in this context. However, the dataset does not contain information on capillary refill time. Blood pressure measurements were frequently not available within the window of tolerance of ± 2 min for data collection. Therefore, these two variables could not be analyzed.

We hypothesize that the association of Hb, mean FiO_2_ and mean SpO_2_ with ΔpCO_2_ may partially be explained by the Haldane effect (40). Reduced oxygen saturation increases the capacity of hemoglobin to bind CO_2_, thereby lowering the fraction of CO_2_ present in dissolved form and reducing blood pCO_2_ for a given total CO_2_ content. This mechanism would be expected to decrease pcCO_2_. Because ΔpCO_2_ was calculated as tcpCO_2_−pcCO_2_, a reduction in pcCO_2_ would increase ΔpCO_2_ if tcpCO_2_ is affected to a lesser extent. As tcpCO_2_ is obtained from heated, locally hyperemic skin and reflects transcutaneous diffusion of CO_2_, the influence of the Haldane effect on tcpCO_2_ is likely less direct than on blood pCO_2_. Therefore, the observed association may be interpreted as a physiological explanation rather than definitive evidence of a causal mechanism. The negative association with Hb may additionally reflect enhanced CO_2_ transport and diffusion dynamics at higher hemoglobin concentrations.

The influence of last tcpCO_2_ and last ΔpCO_2_ on ΔpCO_2_ is likely to be attributable to interindividual factors. As these parameters are directly involved in the calculation of ΔpCO_2_, we did not include them in a multivariable analysis. Parameters not regarded in this study could possibly explain these differences. We therefore believe that tcpCO_2_ dynamics should also be regarded as relevant influence parameters on ΔpCO_2_ at this stage.

The proportional bias observed in the Bland–Altman analysis suggests that disagreement between tcpCO_2_ and pcCO_2_ increases at higher CO_2_ levels. This finding may be clinically relevant because episodes of hypercarbia are often of concern in the group of EPI due to their association with intraventricular hemorrhage. Consequently, caution may be warranted when interpreting tcpCO_2_ values in the hypercapnic range, as the discrepancy between transcutaneous and capillary measurements may become more pronounced. Further studies focusing specifically on higher CO_2_ ranges are needed to determine the clinical significance of this observation.

Prior work demonstrated that machine learning is very effective in predictive accuracy for non-invasive respiratory monitoring (41). Recent studies have shown that continuously acquired noninvasive physiological signals can be integrated into predictive models to estimate arterial blood gas parameters, including arterial pCO_2_ and pH, thereby reducing reliance on repeated invasive sampling (42). Similar approaches have been used for oxygenation assessment, where SpO_2_- and FiO_2_-based models were shown to accurately impute arterial pO_2_/FiO_2_ ratios in mechanically ventilated patients (43). Our findings extend this concept by identifying physiological determinants of the discrepancy between transcutaneous and capillary CO_2_ measurements. The identified parameters may help to establish a more accurate estimate of pCO_2_ in future studies, e.g., by incorporating the variables using machine learning techniques or predictive linear regression models.

### Limitations

Extremely premature birth is very rare, affecting only approximately 0.42% of all neonates. It is therefore difficult to acquire larger study cohorts for data analysis in a monocentric design. There is furthermore a lack of publicly available EPIs datasets which could be used to evaluate potential influence factors on ΔpCO_2_. The sample size included in the analysis is therefore limited. Other studies frequently include more mature infants and can therefore achieve higher sample sizes (32, 44). The validation of the results on external data is desirable to enhance the generalizability of the results.

Multivariable adjustment is important to address potential confounding. In our dataset, the number of independent observational units was limited to 29 individual patients. Because the effective sample size for inference in a mixed-effects framework is determined primarily by the number of independent clusters rather than the total number of measurements, fitting a model with many covariates would risk overparameterization, unstable estimates, and unreliable standard errors. For this reason, we restricted multivariable adjustment to a small number of clinically pre-specified covariates and avoided a fully saturated model including all significant parameters from the univariate models.

Another limitation is the absence of information on surfactant administration, which is frequently conducted during the first postnatal week. It may alter the ventilation-perfusion matching and hereby CO_2_ clearance, which potentially affects both pcCO_2_ and tcpCO_2_ measurements.

Sensor-specific factors such as calibration, sensor drift and shift, change of sensor location and membrane can also affect ΔpCO_2_. As our dataset does not contain information on these parameters, they could not be included in the analysis. It would be interesting to address the influence of sensor location in future studies. Furthermore, the low sensor temperature of 40.2 °C can influence the results. We use this temperature in standard care to decrease the risk for skin burns. Prior studies demonstrated that the use of low sensor temperatures in premature infants is feasible, although it leads to larger ΔpCO_2_ values compared to the standard temperature of 42 °C (45–47). In our study, tcpCO_2_ values would therefore likely have been higher if a higher sensor temperature had been used. We expect that the observed association of vital signs and tcpCO_2_ dynamics on ΔpCO_2_ were not caused by the chosen temperature.

Finally, the type of blood sampling has an impact on the results. On our NICU, umbilical catheters are rarely used in extremely premature infants due to the risk of thrombosis and embolies. Although arterial pCO_2_ constitutes the gold standard for CO_2_ monitoring, blood sampling is predominantly conducted from the capillaries on our ward. pcCO_2_ values are known to exceed arterial pCO_2_ values by approximately 1.5 mmHg (48). As this deviation is only small, we believe that the influence of capillary blood sampling on our results is limited. This is in accordance with the findings of Borenstein-Levin et al., who could not find significant differences in tcpCO_2_ accuracy between different sampling methods (23).

## Conclusion

To the best of our knowledge, this is the first study to identify a variety of candidate influence factors including patient characteristics, vital signs and tcpCO_2_ dynamics on ΔpCO_2_ in EPIs in their first week of life. We found that multiple candidate parameters are associated with higher ΔpCO_2_. The results help to identify possible predictors which can be used to improve the non-invasive estimation of pCO_2_ by developing predictive models in future studies.

## Data Availability

The raw data supporting the conclusions of this article will be made available by the authors, without undue reservation.
